# Effectiveness of XP-Endo Finisher and passive ultrasonic irrigation on intracanal medicament removal from root canals: a systematic review and meta-analysis

**DOI:** 10.1186/s12903-021-01644-7

**Published:** 2021-06-09

**Authors:** Jiani Zhou, Tingjun Liu, Lihong Guo

**Affiliations:** 1 Hospital of Stomatology, Guanghua School of Stomatology, Sun Yat-Sen University, 56 Lingyuanxi Road, Guangzhou, 510055 China; 2 Guangdong Provincial Key Laboratory of Stomatology, Guangzhou, China

**Keywords:** Endodontics, Intracanal medicament removal, Meta-analysis, Passive ultrasonic irrigation, Root canal therapy, XP-endo Finisher

## Abstract

**Background:**

XP-Endo Finisher (XPF) and passive ultrasonic irrigation (PUI) are commonly used in intracanal medicament removal. The effectiveness of these two techniques needs to be compared, and evidence-based research should be conducted.

**Methods:**

A comprehensive literature search was conducted in PubMed, Web of Science, Embase, Cochrane Library, and Google Scholar up to December 20th, 2020. The outcomes of the included trials were pooled into the Cochrane Collaboration’s Review Manager 5.3 software. Cochrane’s risk-of-bias tool 2.0 was applied to assess the risk of bias.

**Results:**

Nine articles were included in this systematic review and processed for data extraction, and eight studies were identified for meta-analysis. In general, the use of PUI showed better medicament removal effectiveness than XPF (odds ratio [OR]: 3.09; 95% confidence interval [CI], 1.96–4.86; *P* < 0.001). PUI was also significantly more efficient than XPF in the apical third (OR: 3.42; 95% CI, 1.32–8.84; *P* = 0.01). For trials using sodium hypochlorite (NaOCl) alone, PUI was also significantly more effective than XPF on intracanal medicaments removal (OR: 5.23; 95% CI, 2.79–9.82; *P* < 0.001). However, there was no significant difference between PUI and XPF when NaOCl and ethylenediaminetetraacetic acid (EDTA) were used in combination (OR: 1.51; 95% CI, 0.74–3.09; *P* = 0.26). In addition, for studies whose intracanal medicament periods were two weeks, the effectiveness of PUI was statistically better than the XPF (OR: 7.73; 95% CI, 3.71–16.07; *P* < 0.001). Nevertheless, for trials whose intracanal medicament time was one week or over two weeks, no differences between the XPF and PUI were found (OR: 1.54; 95% CI, 0.74–3.22; *P* = 0.25) (OR: 1.42; 95% CI, 0.44–4.61; *P* = 0.56).

**Conclusions:**

The meta-analysis is the first study to quantitatively compare the effectiveness of XPF and PUI techniques on intracanal medicaments removal. With rigorous eligibility criteria, the study only included high-quality randomised controlled trials. The study indicated that PUI might be superior over XPF techniques for removing intracanal medicaments from artificial standardized grooves and cavities in the root canal system. The anatomical areas, irrigation protocol, and intracanal medicaments time may influence the cleaning efficacy.

**Supplementary Information:**

The online version contains supplementary material available at 10.1186/s12903-021-01644-7.

## Introduction

It is well known that root canal treatment consists of root canal preparation, disinfection, and obturation procedures [1]. Root canal disinfection is one of the most fundamental stages in endodontic treatment. Complete disinfection can be achieved by using various antimicrobial agents in the form of irrigants and medicaments [2]. Associated with mechanical preparation, chemical disinfection might eliminate infected dentine debris and planktonic bacteria [3]. This procedure is critical for reducing postoperative endodontic pain and promoting long-term healing effects [4, 5]. Intracanal medicaments have been applied widely to enhance disinfection efficacy [6]. Medicaments applications contribute to outstanding cleaning effects, especially for complex anatomic areas in root canal systems, such as the apical deltas and isthmus regions [7]. Intracanal remaining medicaments should be eliminated as much as possible before root canal obturation [8]. Otherwise, the residual medicaments might adhere to the canal wall, interfere with the penetration of endodontic sealers into dentinal tubules, and increase the microleakage of obturation materials that lead to treatment failure in the long term [9–11]. Therefore, the removal of dressings placed in the root canal system before obturation is important for decreasing negative effects on further procedures.

Several studies have so far concluded that none of the applicable techniques was able to completely remove medicament substances from root canals [12–14]. It is urgent to compare the efficacy of various techniques in the removal of a root canal dressing, with the purpose of seeking the most effective irrigation method for clinical applications. Various techniques have been suggested for optimising intracanal medicaments removal, including syringe and needle irrigation (SNI) [15], CanalBrush (CB) [16], passive ultrasonic irrigation (PUI) [6], self-adjusting file (SAF) [17], Endo Activator (EA) [18], laser-activated irrigation (LAI) [19], and XP-endo Finisher (XPF) [20]. However, a consensus on the best method is yet to be achieved. PUI is one of the most universal and well-established irrigation methods [21]. In complex canal anatomic areas, the use of PUI could improve the cleaning effects by cavitation and acoustic micro streaming [13, 22]. The commonly used tip for PUI is a threaded ultrasonic file, and the instrument’s size is matched to the file size based on the International Organization for Standardization [23]. The ultrasonic therapy was considered as the gold standard in the past, and it was thought to be more effective than SAF, SNI, EA, LAI, and CB in removing medicaments from the root canal system [9, 16, 17, 24–26]. However, in recent years, XPF (FKG Dentaire, La Chaux de Fonds, Switzerland) was gradually applied in endodontic treatment. This novel nickel-titanium rotary finishing file could expand at body temperature with high flexibility. The characteristic also contributes to the mechanical cleaning of canal space with complex morphology [27, 28]. Previous literature has analysed the functions of the XPF instrument on removing intracanal medicaments by comparing it with other techniques. Several studies reported that XPF was more effective than SNI on the removal of intracanal medicaments [29–31]. In simulated internal resorption cavities, XPF was superior to SNI, CB, and EA on the medicament removal [32]. XPF was more effective for removing calcium hydroxide residues than the XP-endo Shaper in extracted maxillary central incisors [14]. Moreover, in simulated immature root canals, the cleaning efficacy of XPF on medicaments removal was also better than SNI and EA [33].

A wide array of studies compared the efficacy of PUI with that of XPF, and their results showed clear controversy [26, 30, 33]. A systematic review by Lauritano et al. revealed that it was still unclear whether XPF could outperform PUI in terms of intracanal medicaments removal [34]. To sum up, most researchers regarded the PUI as the gold standard for irrigation in the past. However, XPF is a new instrument with high efficacy. Therefore, assessing the effectiveness of PUI and XPF on intracanal medicaments removal is critical. The present study is the first meta-analysis to make a quantitative comparison between these two techniques, and it provides evidence-based results and offers clinicians a helpful guideline in endodontic therapy.

## Methods

The review was carefully prepared following the Preferred Reporting Items for Systematic Reviews and Meta-Analyses (PRISMA) guidelines [35] and the 2019 Cochrane Handbook [36]. The protocol was registered in the International Prospective Register of Systematic Reviews (PROSPERO) under the number CRD42020199203.

### Focused question

This systematic review and meta-analysis compared the efficacy of XPF with that of PUI on intracanal medicaments removal from root canals. The details of the focused question were as follows: 1. Participants (P): extracted human teeth with intact root canal systems. 2. Intervention (I): the use of XPF alone in removing intracanal medicaments during endodontic therapy. 3. Comparison (C): the use of PUI alone in removing intracanal medicaments during endodontic therapy. 4. Outcome (O): outcome of interest was evaluated using a scoring system described by Lee et al. [37] or van der Sluis et al. [38] according to the quantity of medicaments remained in the root canal systems.

### Search strategy

Two authors (JZ and TL) independently searched for relevant literature up to December 20th, 2020 in four key databases, including PubMed, Web of Science (WoS), Embase, and Cochrane Library. In all databases mentioned above, the following search strategy was used: (passive ultrasonic activation OR passive ultrasonic irrigation OR ultrasonically activated irrigation OR passive ultrasonic agitation OR ultrasonic irrigation OR ultrasonic activation OR ultrasonic agitation OR ultrasonic therapy OR ultrasound OR PUI OR ultrasonic OR ultrasonics) AND (XP-endo Finisher file activation OR XP-endo Finisher file irrigation OR XP-endo Finisher OR XP-endo Finisher file OR XP-endo file OR Xp-endo Finisher OR XP-Finisher rotary file OR XP Endo Finisher OR Finisher file OR XP file OR XP-F OR XPF OR finisher file OR finishing file OR nickel-titanium rotary finishing file OR nickel-titanium file OR rotary file activation OR rotary finishing file OR file activation OR file irrigation). In the PubMed database, publications were sorted by ‘most recent,’ and the publication language was confined to ‘English.’ In the WoS database, the citation indexes were restricted to ‘SCI-EXPANDED.’ In the Embase database, the sources were limited to ‘Embase only,’ and the publication language was restricted to ‘English.’ In addition, the Google Scholar database was manually searched for eligible articles not indexed in the four databases mentioned above. Two authors (JZ and TL) searched each database independently and resolved disagreements by discussing their search results.

### Eligibility criteria

The inclusion criteria were as follows:

1. The study was a randomised controlled trial (RCT) or controlled clinical trial (CCT).
2. The study used extracted human teeth with simulated internal resorption cavities or an artificial standardised groove in root canals.
3. XPF was involved in the experimental group(s) to remove intracanal medicaments from root canals.
4. PUI was involved in the control group(s) to remove intracanal medicaments from root canals.
5. The outcome was evaluated using a scoring system described by Lee et al. [37] or van der Sluis et al. [38].

All specimens were split into two halves longitudinally, and the standardised grooves or resorption cavities were prepared in the root canal walls. Digital photographs of each root canal were taken before medicaments placement and after the removal of medicaments using a microscope and a digital camera. The quantity of medicaments remained in the root canal was scored: score 0 indicated that the root canal was free of medicaments; score 1 demonstrated that less than half of the root canal was covered with medicaments; score 2 indicated that more than half of the root canal was filled with medicaments; score 3 referred to a root canal entirely covered with medicaments.

Reviews, case reports, case series, letters, personal opinions, conference abstracts, book chapters, and animal studies were excluded. Moreover, relevant studies were excluded if the outcomes of interest were not extractable.

### Literature screening and data extraction

All publication records were imported into EndNote X9 software, and duplications were removed. Two authors (JZ and TL) independently screened and assessed studies’ titles, abstracts, and full-texts to identify eligible studies in accordance with the eligibility criteria. Irrelevant studies were excluded after the title and abstract screening. References of all eligible studies were also examined. Two authors (JZ and TL) resolved disagreements by consulting with a senior author (LG).

Data from each included study was extracted and summarised using a predefined data collection form. The relevant items were as follows: first author, year of publication, the number of teeth, intracanal medicaments time, irrigation protocol, cases, and scoring results of the XPF group and the PUI group. For dichotomous statistical analysis, the outcomes of interest were categorised, based on the scoring system, as follows: 1. Success: medicaments were present in not more than half of a single root canal (score 0 or 1). 2. Failure: more than half of single root canal covered with medicaments (score 2 or 3).

### Risk-of-bias assessment and statistical analysis

The Cochrane’s risk-of-bias tool (RoB 2.0) was applied to assess the risk of bias arising from the following five domains: randomisation process, deviations from intended interventions, missing outcome data, measurement of the outcome, and selection of the reported result [39]. Two authors (JZ and TL) independently assessed the risk of bias in each domain following a series of signalling questions, and they evaluated the overall bias of each included trial based on the algorithm described by RoB 2.0 guidelines. If the trial had low risk in all domains, it was judged to have a low risk of overall bias. If the trial had high risk in any domain, it was judged to have a high risk of overall bias. If the trial had low risk or some concerns, with no high risk in any domain, it was considered to have some concerns overall. Disagreements between two assessors (JZ and TL) were resolved by discussion with a senior author (LG).

The outcomes of interest of the included trials were pooled into Review Manager 5.3 software (The Nordic Cochrane Centre, Copenhagen, Denmark). The odds ratio (OR) was calculated to compare the failure rate of the XPF group with that of the PUI group, and the results are reported with 95% confidence intervals (CIs). Subgroup analyses were performed based on different intracanal medicament periods, different irrigation protocols, or different root canal areas that were assessed. Statistical heterogeneity among trials was calculated by a chi-square test (significance: *p* < 0.10) and Higgins index (*I*^2^) [40]. The *I*^2^ statistic indicates the diversity of variation among the eligible studies, and the diversity stems from heterogeneity rather than sampling error. If *I*^2^ was no more than 50%, we would apply the Mantel–Haenszel fixed-effects model of analysis; if *I*^2^ was greater than 50%, we would apply the random-effects model of analysis [41]. All results are displayed as ORs with 95% CIs and are shown in forest plots.

## Results

### Literature selection

**Fig. 1:**
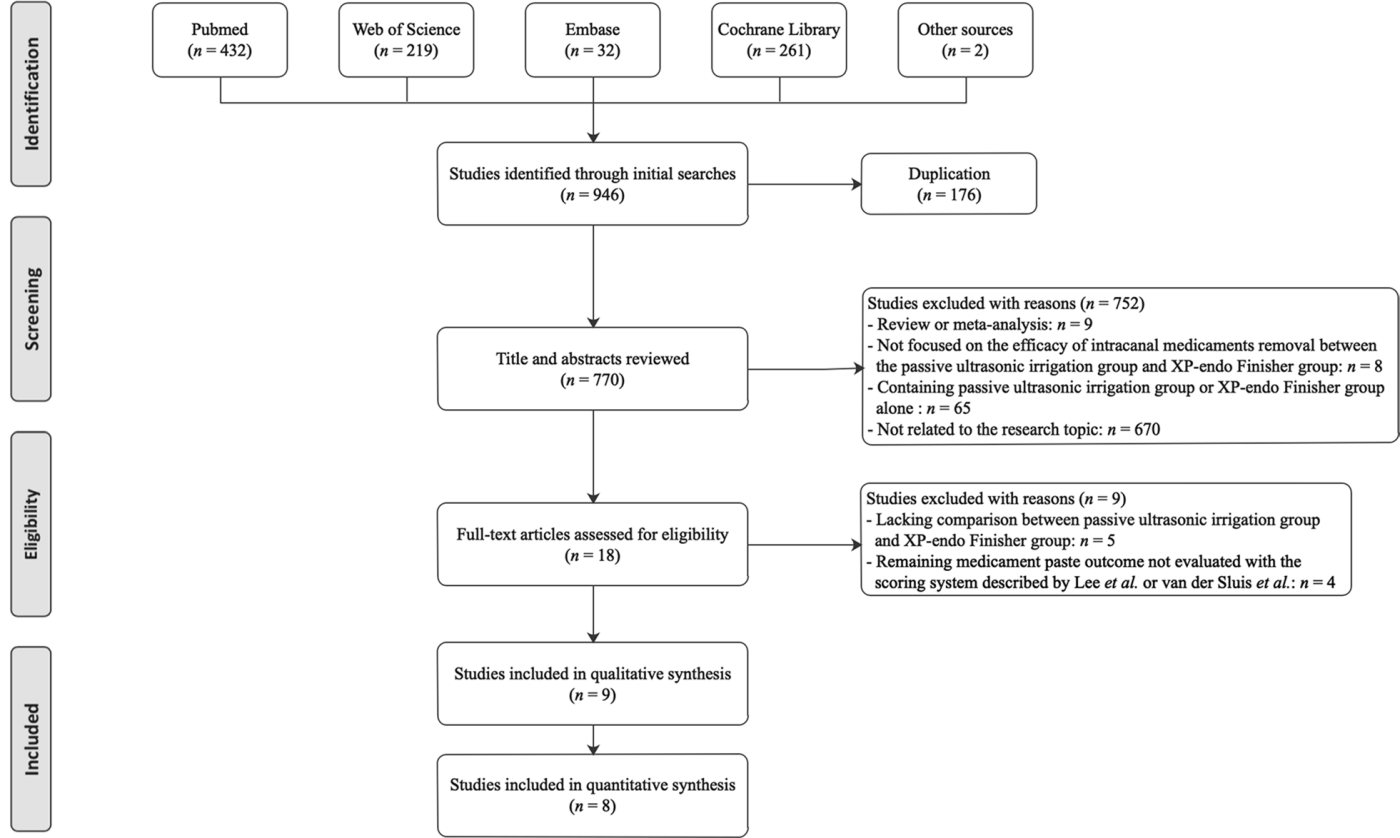
PRISMA flow chart of study selection process

In total, we identified 946 studies initially during the literature search (Fig. 1). After de-duplication, we browsed titles and abstracts of the remaining 770 studies, and we excluded 752 articles. Among the excluded studies, nine were reviews or meta-analysis [42–50], four compared the effectiveness of PUI with XPF on removing hard-tissue debris or smear layer [51–54], three evaluated the efficacy on biofilm or bacteria removal when using the two techniques [55–57], one focused on post-operative pain of root canal treatment using the PUI versus XPF [58]. Moreover, 13 studies did not investigate the effectiveness of XPF on intracanal medicaments removal, 45 articles examined the efficacy of PUI on hard-tissue debris, smear layer, root filling remnants, bacteria, or biofilm removal. In addition, three studies only reported the efficacy of XPF instruments [14, 59, 60], four studies investigated the root filling remnants effects of XP-endo Finisher R instrument [61–64]. The other 670 studies focused on the fields of oral and maxillofacial medical imaging, oral and maxillofacial surgery, periodontology, prosthodontics, and other irrelated research topics. Then, a total of 18 articles underwent a full-text screening. In this phase, nine [14, 20, 65–71] studies were excluded for the following reasons: five [14, 65, 68, 70, 71] lacked a comparison of medicament removal efficacy between the PUI group and XPF group, and four [20, 66, 67, 69] did not evaluate outcomes according to the scoring system described by Lee et al. or van der Sluis et al. Finally, nine articles were included in the present systematic review and processed to data extraction [26, 29–33, 72–74]. Among the nine studies included for qualitative synthesis, one was not appropriate for the quantitative synthesis, because it just presented the minimum and maximum of the scoring results in the PUI and XPF group [26]. Therefore, eight studies were identified for meta-analysis [29–33, 72–74]. References listed in all eligible studies were screened, and no additionally eligible studies were identified.

### Characteristics of the included studies

The characteristics of the eligible publications are shown in Table 1. All articles are published in English. All teeth in each trial are human teeth with straight roots and a single root canal. After the removal of medicament, researchers used the same scoring system to evaluate the specimens’ effects by observing digital photographs [37, 38]. A total of eight trials with 754 specimens (374 in XPF groups and 380 in PUI groups) were analysed in the quantitative synthesis stage [29–33, 72–74]. In each study, the XPF file was employed according to the manufacturer’s instructions. In detail, the file tip was placed 1 mm short of the working length (WL), with a speed of 800 rpm and a maximum torque of 1 N/cm. The working time was 1 min, and the vertical movement distances were 7–8 mm to the full WL. In addition, in each trial included for quantitative synthesis, the ultrasonic tip in the PUI group was placed 1 mm short of the WL and driven by an ultrasonic device with power ranging from 5 to 9. The working time was 1 min. Moreover, there were two studies dividing the standardised artificial grooves into three sections: apical, coronal, and middle section [72, 73]; three trials focused on the apical third of root canals [29, 30, 74]. Furthermore, the intracanal medicament time varied among these studies. Five articles reported that medicaments were stored in the root canal for a week [26, 29, 30, 32, 74], two for two weeks [72, 73], one for a month [33]. Meanwhile, a study contained three kinds of intracanal medicament periods; in detail, the medicaments were stored for 7, 21, and 90 days [31]. Moreover, a study applied both double and triple antibiotic pastes as the intracanal medications [33]. In terms of the apical diameter and taper in the process of preparation, root canal was prepared to size 40/0.04 in six studies, and the preparation diameter was even performed to size 50 in three studies. The adequate apical diameter and taper may provide sufficient space for tips to agitate irrigating agent, and decrease any possible friction. The ideal apical diameter and taper may also add the choice by endodontist for a better performance [26].

**Table 1:** The main characteristics of the included studies

Authors; year	Teeth number	Intracanal medicament time (stored at 37 °C with 100% humidity)	Groups (n)	Irrigants	Evaluation method	XPF group		PUI group	
						Cases	Scoring result	Cases	Scoring result
Gokturk, 2016	105	For 2 weeks	XPF (n = 15) PUI (n = 15)	NaOCl	Stereomicroscope at 20 × magnification	15	Coronal third Score0: 0 Score1: 5 Score2: 10 Score3: 0	15	Coronal third Score0: 1 Score1: 9 Score2: 5 Score3: 0
						15	Middle third Score0: 0 Score1: 3 Score2: 11 Score3: 1	15	Middle third Score0: 0 Score1: 9 Score2: 6 Score3: 0
						15	Apical third Score0: 0 Score1: 1 Score2: 9 Score3: 5	15	Apical third Score0: 0 Score1: 3 Score2: 9 Score3: 3
Gokturk, 2017	105	For 2 weeks	XPF (n = 15) PUI (n = 15)	NaOCl	Stereomicroscope at 20 × magnification	15	Coronal third Score0: 0 Score1: 4 Score2: 11 Score3: 0	15	Coronal third Score0: 7 Score1: 5 Score2: 3 Score3: 0
						15	Middle third Score0: 0 Score1: 4 Score2: 9 Score3: 2	15	Middle third Score0: 7 Score1: 5 Score2: 3 Score3: 0
						15	Apical third Score0: 0 Score1: 0 Score2: 7 Score3: 8	15	Apical third Score0: 1 Score1: 7 Score2: 7 Score3: 0
Keskin, 2017	100	For 1 week	XPF (n = 18) PUI (n = 18)	NaOCl + EDTA	Stereomicroscope at 20 × magnification	36	Score0: 9 Score1: 12 Score2: 11 Score3: 4	36	score0: 16 score1: 11 score2: 5 score3: 4
Uygun, 2017	32	For 1 week	XPF (n = 8) PUI (n = 8)	EDTA	Stereomicroscope at 25 × magnification	16	Apical third Score0: 12 Score1: 3 Score2: 1 Score3: 0	16	Apical third Score0: 14 Score1: 2 Score2: 0 Score3: 0
Wigler, 2017	68	For 1 week	XPF (n = 20) PUI (n = 20)	NaOCl	Microscope at 24 × magnification	20	Apical third Score0: 0 Score1: 2 Score2: 14 Score3: 4	20	Apical third Score0: 0 Score1: 3 Score2: 14 Score3: 3
Keskin, 2018	190	For 7, 21 or 90 days	XPF (n = 20) PUI (n = 20)	NaOCl + EDTA	Stereomicroscope at 30 × magnification	34	7 days Score0: 19 Score1: 15 Score2: 0 Score3: 0	40	7 days Score0: 20 Score1: 18 Score2: 2 Score3: 0
						40	21 days Score0: 11 Score1: 28 Score2: 1 Score3: 0	40	21 days Score0: 17 Score1: 22 Score2: 1 Score3: 0
						40	90 days Score0: 8 Score1: 32 Score2: 0 Score3: 0	40	90 days Score0: 18 Score1: 22 Score2: 0 Score3: 0
Kfir, 2018	80	For 1 week	XPF (n = 18) PUI (n = 18)	NaOCl	Microscope at 24 × magnification	18	Apical third Score0: 0 Score1: 2 Score2: 13 Score3: 3	18	Apical third Score0: 0 Score1: 2 Score2: 13 Score3: 3
Donnermeyer, 2019	90	For 1 week	XPF (n = 20) PUI (n = 20)	NaOCl	Laser scanning microscope at 10 × magnification	20	NA	20	NA
Sarıyılmaz, 2019	180	For 1 month	XPF (n = 20)PUI (n = 20)	NaOCl + EDTA	Stereomicroscope at 10 × magnification	40	DAP Score0: 20 Score1: 16 Score2: 4 Score3: 0	40	DAP Score0: 13 Score1: 26 Score2: 1 Score3: 0
						40	TAP Score0: 23 Score1: 15 Score2: 2 Score3: 0	40	TAP Score0: 19 Score1: 18 Score2: 3 Score3: 0

Abbreviations: XPF, XP-endo Finisher; PUI, passive ultrasonic irrigation; NA, not available; DAP, double antibiotic paste; TAP, triple antibiotic paste

### Risk of bias assessment

The risk of bias analysis is shown in Additional file 1: Figs. S1, S2. All nine included studies were judged to raise some concerns overall. Although all the included trials mentioned the term ‘randomly’ to describe the method of dividing the samples, specific randomisation methods were not mentioned in eight trials. Only one manuscript described that it carried out a random sequence generated by flipping coins [29]. Meanwhile, none of the included literature performed allocation concealment. Therefore, nine studies (100%) showed some concerns about randomisation process bias. There were no deviations from intended interventions, missing outcome data, or reporting bias in all the eligible trials. Concerning the bias of outcome measurement, all the included studies evaluated the outcomes using the four-grade scoring system described by Lee et al. [37] or van der Sluis et al. [38]. In addition, in all included studies, the amount of the remaining medicament was independently scored by two calibrated and blinded doctors. Consequently, all eligible studies were deemed to have a low risk of bias in the measurement of the outcome.

### Quantitative synthesis

**Fig. 2:**
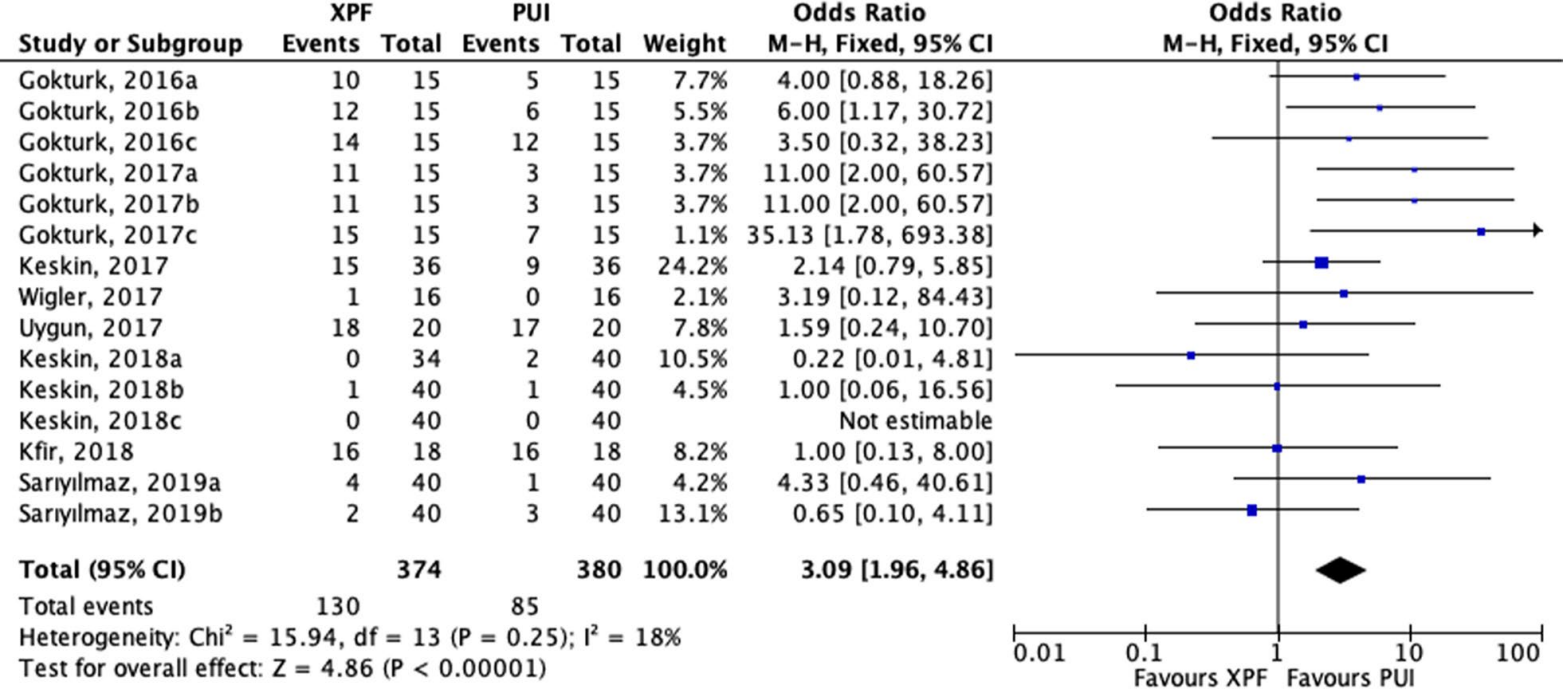
Forest plot and meta-analysis. Forest plots display the odds ratio related efficacy of intracanal medicament removal: XPF versus PUI. Event indicateds the number of failures. XPF, XP-Endo Finisher; PUI, passive ultrasonic irrigation; CI, confidence interval; M-H, Mantel Haenszel test

**Fig. 3:**
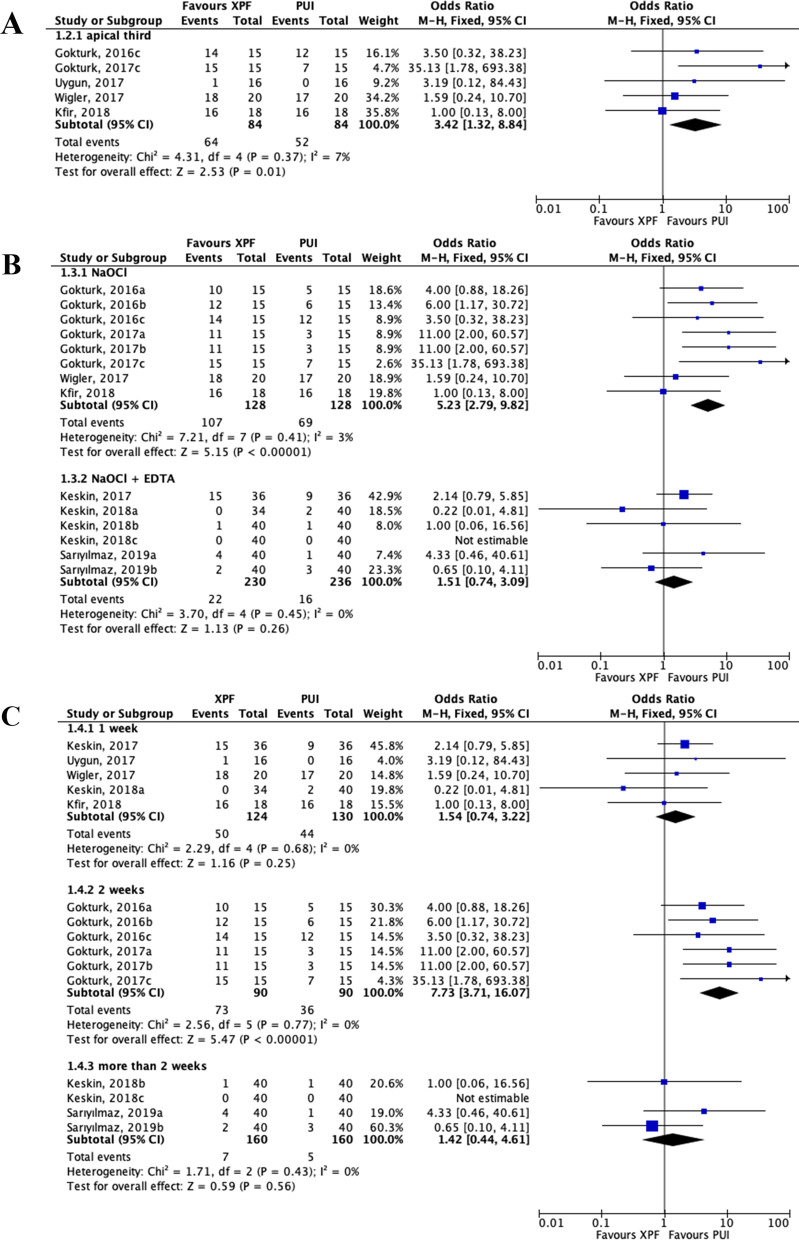
Forest plot of the subgroup analyses. **a** Anatomical areas limited to canal third; **b** irrigants type; **c** intracanal medicament time. The odds ratio depicted efficacy of intracanal medicament removal: XPF versus PUI. Event indicateds the number of failures. XPF, XP-Endo Finisher; PUI, passive ultrasonic irrigation; CI, confidence interval; M-H, Mantel Haenszel test

Outcome data from a total of 754 teeth or root halves were collected to calculate the overall removal efficacy between the XPF and PUI group. The failure rate of removal intracanal medicaments was 34.8% (130/374) in the XPF group and 22.4% (85/380) in the PUI group. Since the heterogeneity was low across the trials, we applied a fixed-effects model (χ^2^ = 15.94, degrees of freedom = 13, *P* = 0.25, *I*^2^ = 18%; Fig. 2). The overall effect showed that PUI had a greater medicaments removal effectiveness than XPF (OR: 3.09; 95% CI, 1.96–4.86; *P* < 0.001; Fig. 2). Additionally, a subgroup analysis also indicated that PUI was significantly more effective than XPF in terms of removing medicaments in the apical third (OR: 3.42; 95% CI, 1.32–8.84; *P* = 0.01; Fig. 3a). Furthermore, we carried out a subgroup analysis based on irrigants protocols. For trials using NaOCl alone, data showed a significantly better intracanal medicaments removal rate in the PUI group than XPF (OR: 5.23; 95% CI, 2.79–9.82; *P* < 0.001; Fig. 3b). However, for studies combining NaOCl and EDTA as canal irrigants, the forest plot did not demonstrate significant differences between PUI and XPF group (OR: 1.51; 95% CI, 0.74–3.09; *P* = 0.26; Fig. 3b). Nonetheless, concerning the intracanal cleaning effectiveness, a better outcome trend was found in PUI than XPF group. There was no heterogeneity revealed among these data (χ^2^ = 3.70, degrees of freedom = 4, *P* = 0.45, *I*^2^ = 0%; Fig. 3b). Moreover, the subgroup analysis based on intracanal medicament time was conducted. For trials whose intracanal medicament time was two weeks, the result showed that medicaments removal effectiveness of PUI was statistically better than that of XPF (OR: 7.73; 95% CI, 3.71–16.07; *P* < 0.001; Fig. 3c). Nevertheless, for trials whose intracanal medicament time was one week, no differences between the XPF and PUI group were found (OR: 1.54; 95% CI, 0.74–3.22; *P* = 0.25; Fig. 3c). For trials whose intracanal medicament time was over two weeks, no differences were observed, either (OR: 1.42; 95% CI, 0.44–4.61; *P* = 0.56; Fig. 3c).

## Discussion

One of the main goals of root canal disinfection is to eliminate microorganisms [75]. However, it is feared that the solubility of residual medicaments in tissue fluids might facilitate bacterial proliferation, because remnants might prevent sealers adaption and leave voids in the filling dentine interface [6, 76]. Several studies have focused on improving the removal of intracanal medicaments during endodontic treatment process, but none of the activation regimens can render the root canals completely free of dressing material [12, 65, 77]. Numerous studies have suggested the superiority of XPF and PUI over other instruments on intracanal medicaments removal [29, 78]. However, a consensus on which approach is better remains to be reached.

### Summary of the main results

This is the first meta-analysis to quantitatively analyse the effect of XPF versus PUI on cleaning residual medicaments in the root canal system. In the present meta-analysis, results suggested that, in general, protocols using PUI were more effective than protocols using XPF in removing medicaments from single straight root canals. The anatomical areas, irrigation protocol, and intracanal medicaments time may influence the cleaning efficacy. Concerning anatomy area on the apical third of root canals, PUI operated superiorly over XPF. In addition, XPF had higher requirements for flushing agents than PUI. XPF might require combining NaOCl and EDTA as the irrigants for performing more effectively.

### Overall completeness and applicability of evidence

In the present meta-analysis, a comprehensive literature search was conducted using multiple databases. In addition to the four key databases, the Google Scholar database and the reference lists of included trials were searched manually. Meanwhile, all included trials were performed by experienced operators in endodontology institutions in college or specialised clinical centres. When trials were operated in different clinical settings, results might change to some extent. In addition, the irrigation protocol of the XPF group was employed according to the manufacturer’s instructions. The PUI group was also performed utilising an ultrasonic tip with a specific placed site, total activation time, and power range. The efficacies of XPF and PUI on medicament removal might be affected by different operation protocols.

### Potential bias in the review process

Seven of the eight included studies did not clarify the randomisation methods in detail, and none of the included trials performed allocation concealment. In addition, considering that ultrasonic file tips have various appearances with XPF file, it was impractical to blind the operators when using instruments in XPF and PUI groups. Lacking the clarification of randomisation and allocation concealment may exaggerate the estimates of intervention effect and contribute to the bias of randomisation process [39]. A high-quality RCT should properly carry out and elaborate on the process of randomisation and allocation concealment, and future trials related to the research topic are encouraged to conduct the process more rigorously. In all included articles, bias arising from intended intervention deviations, measurement of the outcome, missing outcome data, and reporting bias were regarded as ‘low risk.’ All eligible trials reported that evaluators were blinded to group allocation and evaluated the results using the same four-grade scoring system. There was no clear bias arising from the measurement of the outcome. Moreover, bias due to missing outcome data was not observed in the include studies. Regarding the reporting bias, the results in all eligible studies were reported in accordance with the pre-specified outcome assessment criteria. As a result, the reporting bias was avoided in all trials. Overall, bias in all domain might be minimised in future RCTs.

### Agreements and disagreements with other studies or reviews

In recent years, studies on the cleaning effects of instruments in the root canal systems attract more and more attention [79–81]. However, the experimental conditions were difficult to standardise in the complex curved canals, because the range of length, radius, curvature, and isthmus of canals could affect the experimental results [82]. The clinical trials that compared the cleaning capacities of PUI and XPF in the curved canals were scarce and not related to our research topic [57]. Merely four studies focused on the effectiveness of calcium hydroxide removal using PUI in the curved canals, and they did not compare it with XPF [77, 82–84]. Moreover, two trials researched the cleaning efficacy of XPF instrument, but they did not focus on the intracanal medicaments removal effects [85, 86]. So far, the intracanal medicaments removal efficacy of PUI and XPF has not been compared in curved root canals, hence all trials included in the meta-analysis were conducted only in the single straight root canals.

The present quantitative synthesis illustrated a better effectiveness of PUI than XPF on intracanal medicaments removal in single straight root canals, which can be explained by the following reasons. The file size, working mechanism, and tip insertion position of PUI could benefit its effects in single straight root canals. First, tip size of the PUI file is matched to the intracanal diameters, and the final preparation size of the physiological foramen. Before the process of root canal disinfection, the single straight root canals were usually prepared up to size 40/0.04, which reached a balance among cleaning effectiveness, anatomical enlargement, and the apical accident risk [32, 74, 79]. However, XPF is a size 25, and nontapered instrument, thereby the size of XPF might be less matched to the foramen size than PUI [28]. Second, the working mechanism of the PUI depends on higher velocity and irrigant stream, along with a thermal effect and the transmission of energy [87, 88]. It contributes to the cleaning effects on the large straight canal space by flowing in the apical-to-coronal direction [55, 89]. A study published by Sabins et al*.* found that a working time of 30 s was sufficient for the PUI to exert a higher cleaning effect than SNI [90]. However, in the large canal space, one minute was probably not sufficient for the XPF to remove intracanal medicaments efficiently [29, 74]. Meantime, Kfir et al*.* and Wigler et al. also worried that the contact time between the XPF file and the groove in straight root canals was too short [29, 74]. Third, the situation of the tip placement would impact the irrigation effects in endodontic treatment [91]. Uzunoglu et al*.* reported that PUI might decrease the amount of irrigant extruded through the apex [92]. In teeth with open apex, Peeters et al*.* also revealed that use of PUI during final irrigation procedures barely resulted in apical extrusion of NaOCl in endodontic therapy [93]. One explanation is that with the insertion depth of the ultrasonic tip becoming deeper, the amounts of debris and irrigants extrusion would also increase [94]. Therefore, from the standpoint of efficiency and safety, the use of PUI was better than XPF in intracanal medicaments removal in single straight root canals.

However, the cleaning effectiveness of PUI might be reduced in the complex curved root canals. Amato et al. compared the ultrasonic action efficiency between straight and curved root canals, founding that the dental debris removal efficacy of PUI could be decreased in curved root canals [95]. One possible explanation was that touching the curved root canal wall would reduce the action of ultrasonic inevitably [95]. Compared with the PUI files, XPF could expand more flexibly and have better fracture resistance, making it better adapt to the irregular anatomy of curved canals [86]. In detail, the XPF could change to a unique spoon shape and adapt three-dimensionally to the root canal morphology at body temperature [55]. Meanwhile, the file has good resistance to fatigue and high stress, which is of particular importance for irrigation in curved root canals. Vaz-Garcia et al*.* concluded that XPF instruments performed better when compared its cyclic fatigue with that of the other anatomic finishing file, XP-Clean instruments [96]. However, ultrasonic tips might fracture during the endodontic shaping process [97]. In addition, Song et al. suggested that pre-curved files removed calcium hydroxide more effectively than the none-pre-curved files in curved root canals [83]. However, during the PUI procedure, pre-curving the ultrasonic file to fully adapt to the curved root canals is challenging. The tip of the PUI file unable to fully extend into the apical position because straight-line access is difficult to build [98]. Therefore, when the tip of the XPF file showed a unique spoon-shape and flexibly extended into the complex apical thirds in the curved root canal, the effects of intracanal medicaments removal might be higher than using the PUI file [97]. Further studies are encouraged to investigate the efficacy of instruments on intracanal remnants removal in the curved root canals.

In addition, as the subgroup analyses showed, in teeth with single straight root canals, anatomical areas, irrigant protocols, and intracanal medicament time might influence the overall intracanal medicaments removal effectiveness.

Consistent with some previous studies [72, 73], PUI were superior to XPF for removing medicaments from the apical thirds of single straight root canals. A systematic review published by Yaylali et al*.* showed that PUI was superior over SNI and EA for removing calcium hydroxide from the root canal apical third area [48]. It is more difficult for the irrigation techniques to fully contact with the canal wall because the apical thirds have more lateral canals, apical ramifications, and isthmus than the coronal and middle thirds [99]. In addition to the complex anatomical factors, the phenomenon of vapor lock also prevented the irrigant solutions to penetrate into apical thirds [100]. PUI was effective in eliminating vapor lock during endodontic irrigation in the apical third of the root canals [101]. Donnermeyer et al*.* also reported that PUI was significantly better than XPF in the removal of medicaments from the apical thirds [26].

In addition, the meta-analysis suggested that PUI performed more effectively than XPF when NaOCl was used as the only flushing agent. NaOCl and EDTA have been the most commonly used irrigating solutions, with the function of dissolving organic substances, killing microbes, and cooling files [87, 102]. Lee et al*.* reported that the combined use of ultrasound and NaOCl led to synergistic effects on reducing bacteria on steel and iceberg lettuce [103]. Therefore, using NaOCl as the irrigant might enhance the working efficacy of PUI. However, when NaOCl and EDTA were used in combination, the cleaning efficacy of the PUI and XPF was similar. Azimian et al*.* assessed the efficacy of XPF on the removal of smear layer and residual debris [104]. They suggested that the synergistic effect of XPF and EDTA was beneficial for better root canal cleaning effects [104]. With the assistance of EDTA, XPF could achieve similar cleaning effectiveness with PUI on the removal of intracanal medicaments. In addition, the concentrations of irrigants and operation temperature might also influence the cleaning effectiveness, but the specific mechanisms are still unclear [105].

In past studies, intracanal medicament time ranged from one week to several months [106, 107]. Concerning the studies we included, Keskin et al*.* indicated that the intracanal time of the TAP could not affect the removal effectiveness of PUI [31]. However, considering that the flushing agents among researches might be different, whether the intracanal medicament period would affect the overall comparison still remains to be clarified. Moreover, the present meta-analysis included four different interval times for a more comprehensive comparison and quantitative analysis. The results of subgroup analyses might provide clinicians with more information about the effects of PUI and XPF on medicament removal efficacy.

### Strengths and limitations

The present systematic review and meta-analysis had several advantages. First, two authors searched key databases independently using an adequate searching strategy. It provided a precise screen range and improved the possibility of generalising the outcomes [108]. Second, the detailed inclusion and exclusion criteria were defined beforehand. We focused on the process of intracanal medicaments removal and excluded articles comparing the efficacy of removing other substances in endodontic treatment, such as smear layer, bacteria, and organic tissue. It should be noted that these listed substances were cleaned in different periods of endodontic therapy. The files in different preparation or disinfection process might play different key roles [55]. Third, only RCTs and CCTs, which are regarded as the highest level of evidence, were included in our quantitative analysis [109]. Moreover, prior protocol registration and subgroup analysis were also the strengths of the current meta-analysis [110, 111]. Different root canal area, irrigation protocols, or intracanal medicament periods are potential factors affecting the heterogeneity. To our knowledge, the meta-analysis is the first study to quantitatively compare the effectiveness of XPF with that of PUI techniques on intracanal medicaments removal.

Several limitations exist in the present meta-analysis. First, language was limited to English during the literature screening process, and this might increase the possibility of reporting bias. When compared to non-English-language journals, English-language journals are more likely to publish positive results [89]. Second, because the case number of some studies was small, the statistical power might be reduced. Third, in most of the included trials, the description of RCT was too simple, and it would influence the quality of studies. Accordingly, improving the quality of related RCTs is advocated.

## Conclusions

The meta-analysis is the first study to quantitatively compare the effectiveness of XPF with that of PUI techniques on intracanal medicaments removal. The current study contained a comprehensive literature search strategy, the detailed eligibility criteria, a prior protocol registration and high level of evidence. It indicated that neither XPF nor PUI could completely remove medicament substances from root canals. In addition, PUI might be superior over XPF protocols for removing medicaments from the single straight root canal area. However, the intracanal medicaments removal efficacy of PUI and XPF has not been compared in curved root canals, PUI probably has more limitations than XPF in this anatomical situation. Clinicians are expected to explore other candidate agitation techniques in curved root canals. The review provided evidence-based results and might build a useful guideline for clinical application. Further studies should seek an irrigation device that could render the root canal free of medication completely. What is more, to further assess the efficacy of XPF and PUI techniques in intracanal medicament removal, more large-scale and high-quality RCTs will be warranted in the future.

## Supplementary Information

**Additional file 1.** **Fig S1.** Risk of bias summary. **Fig S2.** Risk of bias graph. Assessment is presented as percentages across all included studies.

## Data Availability

All data generated or analysed during this study are included in this published article.

## Abbreviations

XPF: XP-Endo Finisher
PUI: Passive ultrasonic irrigation
OR: Odds ratio
CI: Confidence interval
NaOCl: Sodium hypochlorite
EDTA: Ethylenediaminetetraacetic acid
SNI: Syringe and needle irrigation
CB: CanalBrush
SAF: Self-adjusting file
EA: Endo Activator
LAI: Laser-activated irrigation
PRISMA: Preferred Reporting Items for Systematic Reviews and Meta-Analyses
PROSPERO: International Prospective Register of Systematic Reviews
WoS: Web of Science
RCT: Randomised controlled trial
CCT: Controlled clinical trial
RoB: Risk-of-bias
WL: Working length
