# Supplementary material for: Effectiveness of XP-Endo Finisher and passive ultrasonic irrigation on intracanal medicament removal from root canals: a systematic review and meta-analysis
Source: BMC Oral Health. 2021 Jun 9;21:294. doi: 10.1186/s12903-021-01644-7 (PMC8191128; doi:10.1186/s12903-021-01644-7)

**Supplementary figure legends**

**Fig S1. Risk of bias summary.**

**Fig S2. Risk of bias graph.**

Assessment is presented as percentages across all included studies.

Fig. S1


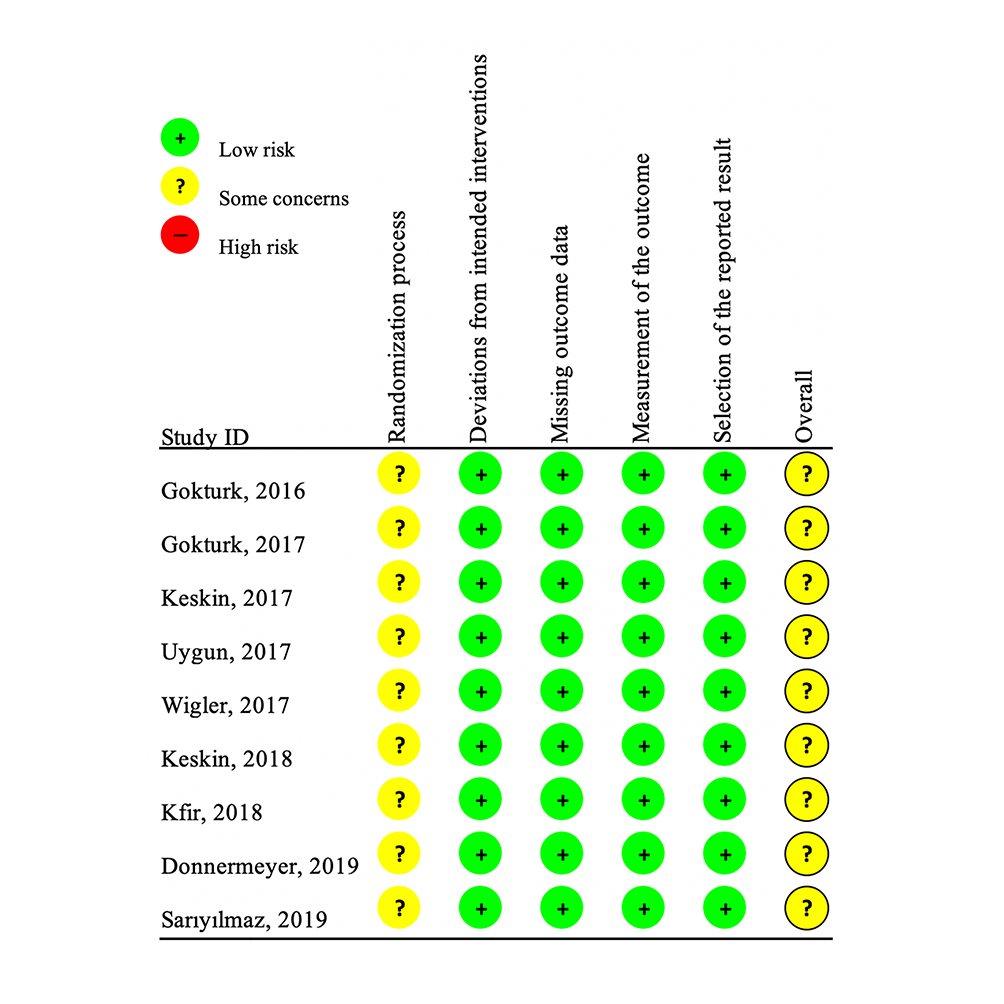


Fig. S2


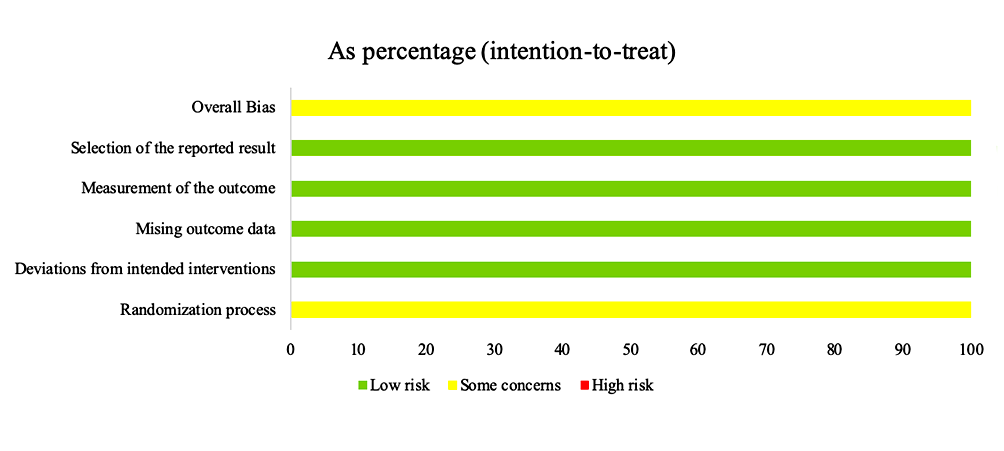

Supplement: Supplementary file 1 — Additional file 1. Fig S1. Risk of bias summary. Fig S2. Risk of bias graph. Assessment is presented as percentages across all included studies. [file 12903_2021_1644_MOESM1_ESM.docx]
